# First Total Synthesis and Biological Screening of a Proline-Rich Cyclopeptide from a Caribbean Marine Sponge

**DOI:** 10.3390/md14120228

**Published:** 2016-12-15

**Authors:** Rajiv Dahiya, Sunil Singh, Ajay Sharma, Suresh V. Chennupati, Sandeep Maharaj

**Affiliations:** 1 Laboratory of Peptide Research and Development, School of Pharmacy, Faculty of Medical Sciences, The University of the West Indies, St. Augustine, Trinidad and Tobago; Sandeep.Maharaj@sta.uwi.edu; 2 Department of Pharmacy, Mewar University, Gangrar, Chittorgarh, Rajasthan 312901, India; 3 Department of Pharmacy, College of Health Sciences, Mizan-Tepi University, Mizan Teferi 5140, Ethiopia; ajaysharmapharma1979@gmail.com; 4 Department of Pharmacy, College of Medical and Health Sciences, Wollega University, P.O. Box 395 Nekemte, Ethiopia; sureshchennupati@rediffmail.com

**Keywords:** stylissamide G, cycloheptapeptide, *Stylissa caribica*, peptide synthesis, macrocyclization, pharmacological activity, marine sponge

## Abstract

A natural heptacyclopeptide, stylissamide G (**7**), previously isolated from the Bahamian marine sponge *Stylissa caribica* from the Caribbean Sea, was synthesized via coupling of the tetrapeptide l-phenylalanyl-l-prolyl-l-phenylalanyl-l-proline methyl ester with the tripeptide Boc-l-leucyl-l-isoleucyl-l-proline, followed by cyclization of the linear heptapeptide fragment. The structure of the synthesized cyclooligopeptide was confirmed using quantitative elemental analysis, FT-IR, ^1^H NMR, ^13^C NMR and mass spectrometry. Results of pharmacological activity studies indicated that the newly synthesized cycloheptapeptide displayed good anthelmintic potential against *Megascoplex konkanensis*, *Pontoscotex corethruses* and *Eudrilus eugeniea* at 2 mg/mL and in addition, potent antifungal activity against pathogenic *Candida albicans* and dermatophytes *Trichophyton mentagrophytes* and *Microsporum audouinii* at a concentration of 6 μg/mL.

## 1. Introduction

Marine natural products with unique structural features and pronounced biological activities continue to provide leading structures in the search for new drugs from nature [1]. Invertebrates such as sponges, tunicates and mollusks have so far provided the largest number of marine-derived secondary constituents. Marine natural product research has spawned several drugs and many other candidates, some of which are the focus of current clinical trials [2]. In spite of large numbers of therapeutic molecules available for human health care, the thrust for safer and effective medicines is increasing. Among natural products, cyclopolypeptides are a unique group of bioactive compounds with interesting pharmacological and biochemical properties which occur mainly in marine sponges and higher plants [3,4]. Detailed investigation of structures and biological potential of naturally occurring cyclooligopeptides suggests that there are cyclic peptide molecules (as reported in literature) with structural patterns containing two proline units separated by one or more phenylalanine units, and these have shown antimicrobial as well as anthelmintic effects. e.g., the proline-rich cyclic heptapeptide (hymenamide E) and cyclotetrapeptide [5,6].

Recently, marine sponge-derived cyclooligopeptides have received the attention of researchers and scientists, as they exhibit a broad range of pharmacological activities, viz. antitubercular activity [7], cytotoxic activity [8], anti-inflammatory activity [9,10], HIV-inhibitory activity [11,12], chymotrypsin-inhibitory activity [13], antimicrobial activity [14] etc. Moreover, peptides (linear and cyclic) are among some of the secondary metabolites isolated from the sponge-associated bacteria (e.g., theopalauamide [15], nazumamide A [16], cycloligopeptides [17], etc.) but these have been found to have no effect on the symbiotic bacteria in sponges. Rather, bacteria provide their hosts with the products of their metabolism, thereby granting the sponge access to bacteria-specific traits such as autotrophy, nitrogen fixation and nitrification. In addition, sponge-associated bacteria process metabolic waste compounds, stabilize the sponge skeleton and provide protection against UV radiation. The most prominent example of sponge bacterial symbiosis is the involvement of bacteria in the production of bioactive metabolites that have a role in defense [18].

A natural cyclic heptapeptide, stylissamide G, was isolated from the extracts of a frozen marine sponge tissue in the Caribbean Sea and characterized by integrated spectroscopic methods [19]. Other natural peptides isolated from marine organisms such as callyaerin A, eudistoamide A, B and wewakpeptin A–D, contain two continuous ‘Pro’ units in a cyclic chain with ‘Leu’/‘Val’/‘Thr’ adjacent to ‘Pro’ units [20,21,22,23]. In comparison, marine sponge-derived heptacyclopeptide stylissamide G, with its characteristic pattern of ‘Pro’ and ‘Phe’ units in repeated manner i.e., two ‘Pro’ units separated by a ‘Phe’ unit, was of particular interest in the present study. Keeping in view the broad range of pharmacological activities possessed by natural cyclopolypeptides [24,25], including the biological potential of other stylissamides (e.g., inhibitory activity towards protein translation/synthesis by Stylissamide A [26], and inhibitory activity against migration of HeLa cells by Stylissamide X [27]), the present investigation was directed toward the synthesis and structure elucidation of stylissamide G by using a solution-phase technique to obtain a natural cyclooligopeptide in the quantitative yield in the laboratory. This study was in continuation of the efforts of our research group for the total synthesis of natural bioactive cyclic peptides of marine and plant origin [28,29,30,31,32,33,34,35,36,37,38,39,40,41,42,43,44,45,46,47,48,49,50,51,52,53]. Keeping in mind characteristic pattern of ‘Pro’ and ‘Phe’ units in its structure, the marine sponge tissue-derived cyclopolypeptide was further subjected to antibacterial, antifungal and anthelmintic screening.

## 2. Results

### 2.1. Synthesis

The solution-phase technique was selected for heptacyclopeptide synthesis, which includes chemistry involving disconnection strategy. In the present investigation, pentafluorophenol (*pfp*) was used for esterification and cyclization during the synthesis of cyclopeptide **7** from the linear peptide unit **6**, affording compound **7** in 72%–86% yield utilizing pyridine/*N*-methylmorpholine (NMM)/triethylamine (TEA) as base. There are previous literature reports on the synthesis of cyclic octapeptide stylissamide X and cyclic heptapeptide stylissamide A by a combination of solid-phase and solution-phase techniques [54,55]. In the present study, the peptide units were prepared by the Bodanszky method with certain modifications [56]. Boc_2_O was used to protect the amino group of l-amino acids. The carboxyl group of l-amino acids was protected by esterification with methanol utilizing SOCl_2_.

Trifluoroacetic acid (CF_3_COOH) was used to remove the Boc group and the ester group was removed by alkaline hydrolysis with lithium hydroxide (LiOH). To avoid any possibility of racemization, 1-Hydroxybenzotriazole (HOBt) was utilized in all the coupling reactions.

**Figure 1:**
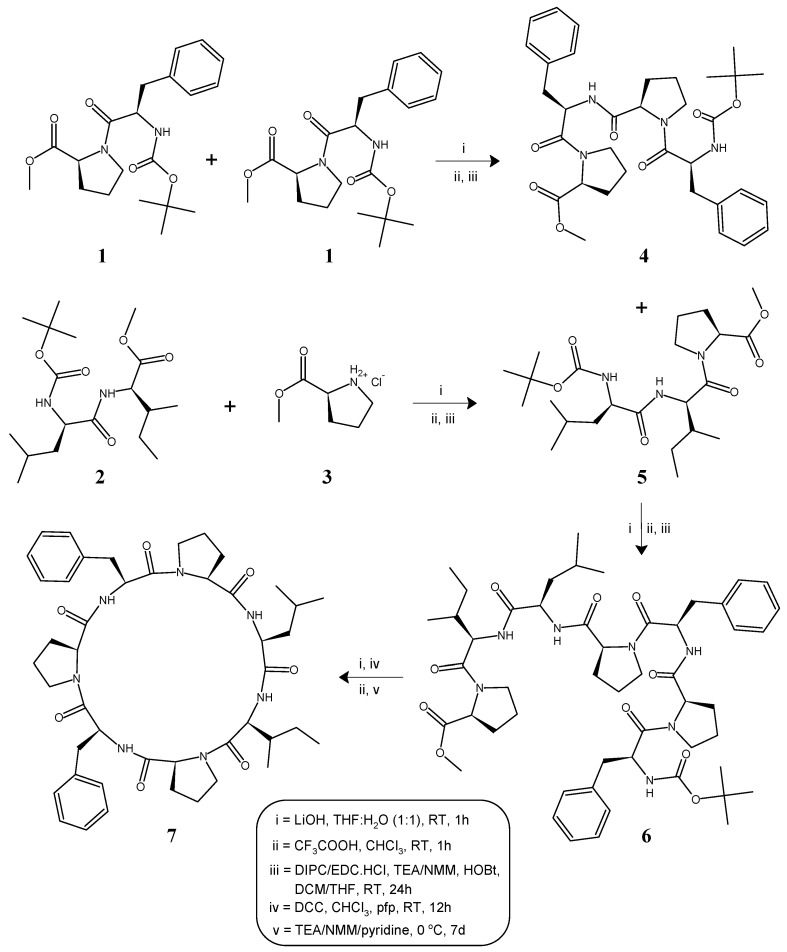
Synthetic route for heptacyclopeptide, stylissamide G (**7**).

The heptacyclopeptide molecule was split into two dipeptide units: Boc-l-Phe-l-Pro-OMe (**1**) and Boc-l-Leu-l-Ile-OMe (**2**) and a single amino acid unit: l-Pro-OMe·HCl (**3**). Dipeptide units (**1**, **2**) were prepared by coupling of Boc-amino acids like Boc-l-Phe-OH and Boc-l-Leu-OH with corresponding amino acid methyl ester hydrochlorides such as l-Pro-OMe·HCl and l-Ile-OMe·HCl. After deprotection at the carboxy terminal by alkaline hydrolysis using LiOH, one unit of dipeptide **1** was coupled with another unit of dipeptide **1**, deprotected at the amino terminal by treatment with CF_3_COOH, to obtain the tetrapeptide unit Boc-l-Phe-l-Pro-l-Phe-l-Pro-OMe (**4**). Similarly, dipeptide **2** deprotected at the carboxyl terminal using LiOH was coupled with the amino acid unit l-Pro-OMe·HCl (**3**), to obtain the tripeptide unit Boc-l-Leu-l-Ile-l-Pro-OMe (**5**). The carboxyl group of tripeptide **5** was removed by alkaline hydrolysis and the deprotected peptide was coupled with tetrapeptide **4**, deprotected at the amino terminal using CF_3_COOH, by utilizing the two different carbodiimides *N*,*N*′-diisopropylcarbodiimide/*N*-(3-dimethylaminopropyl)-*N*′-ethylcarbodiimide hydrochloride (DIPC/EDC·HCl), to obtain the linear heptapeptide unit Boc-l-Leu-l-Ile-l-Pro-l-Phe-l-Pro-l-Phe-l-Pro-OMe (**6**). The methyl ester group of the linear peptide fragment was replaced by the pentafluorophenyl (*pfp*) ester group. The Boc group of the resulting compound was removed using CF_3_COOH and the deprotected linear fragment was then cyclized by keeping the whole contents at 0 °C for 7 days in the presence of catalytic amounts of TEA or NMM or pyridine to obtain cyclic product **7**. The structure of the newly synthesized cyclopolypeptide, as well as that of the intermediate di/tri/tetra/heptapeptides were confirmed by FT-IR, ^1^H NMR spectroscopy and elemental analysis. In addition, mass spectra and ^13^C NMR spectroscopy were recorded for the linear and cyclic heptapeptides. The synthetic pathway for the newly synthesized heptacyclopeptide is shown in Figure 1.

### 2.2. Pharmacology

The anthelmintic activity results for linear and cyclic heptapeptides (**6**, **7**) against three earthworm species *Megascoplex konkanensis*, *Pontoscotex corethruses* and *Eudrilus eugeniea* at 2 mg/mL concentration using modified Garg’s method [57] are compiled in Table 1. Moreover, antimicrobial activity results for newly synthesized linear and cyclic heptapeptides (**6**, **7**) against the four bacteria *Bacillus subtilus*, *Staphylococcus aureus*, *Pseudomonas aeruginosa* and *Klebsiella pneumonia*, cutaneous fungi *Microsporum audouinii* and *Trichophyon mentagrophytes*, and diamorphic fungi *Candida albicans* and *Aspergillus niger* using modified Kirby-Bauer disk diffusion method [58], are tabulated in Table 2.

## 3. Discussion

The synthesis of cyclooligopeptide **7** was accomplished with 86% yield, and pyridine proved to be an effective base for cyclization of the linear heptapeptide unit. Cyclization of the linear peptide fragment was supported by the disappearance of absorption bands at 1745, 1273 and 1387, and 1369 cm^−1^ (C=O_str_, C–O_str_, ester and C–H_def_, *tert*-butyl groups) in IR spectra of compound **7**. The formation of the cyclopeptide was further confirmed by the disappearance of singlets at 3.62 and 1.55 ppm corresponding to three protons of the methyl ester group and nine protons of the *tert*-butyl group of Boc in the ^1^H NMR spectrum, and the disappearance of the singlets at 153.4, 79.6 and 53.3, 28.2 ppm corresponding to carbon atoms of ester and *tert*-butyl groups in the ^13^C NMR spectrum of compound **7**. Furthermore, the ^1^H NMR and ^13^C NMR spectra of the synthesized cyclic heptapeptide showed characteristic peaks confirming the presence of all the 61 protons and 45 carbon atoms. The pseudomolecular ion peak (M + 1)^+^ appears at *m/z* = 813, corresponding to the molecular formula C_45_H_61_N_7_O_7_ in the mass spectrum of **7** along with other fragment ion peaks resulting from cleavage at ‘Pro-Phe’, ‘Ile-Leu’, ‘Pro-Ile’, ‘Phe-Pro’ and ‘Leu-Pro’ amide bonds (Figure S1). In addition, the presence of the immonium ion peaks at *m/z* = 120 (Phe), 86 (Leu/Ile) and 70 (Pro) further confirmed all the amino acid moieties in the cyclopeptide structure. Furthermore, the elemental analysis of cyclopeptide **7** afforded values with tolerance of ±0.02 strictly in accordance with the molecular composition.

Comparison of antifungal activity data suggested that cyclooligopeptide **7** possessed potent bioactivity against dermatophytes *M. audouinii*, *T. mentagrophytes* and pathogenic fungi *C. albicans* with MIC values of 6 μg/mL when compared to the reference drug griseofulvin. From the analysis of anthelmintic activity data, it is observed that cyclopeptide **7** displayed remarkable activity against all three earthworm species *M. konkanensis*, *P. corethruses* and *E. eugeniea*, in comparison to standard drug mebendazole. Moreover, a moderate level of activity was observed against the Gram-negative bacteria *P. aeruginosa* and *Klebsiella pneumonia* for the newly synthesized cyclopeptide, in comparison to the standard drug gatifloxacin. However, compound **7** displayed no significant activity against either Gram-positive bacteria or *Aspergillus niger*. In addition, the analysis of the pharmacological activity data revealed that heptacyclopeptide **7** displayed a higher bioactivity against pathogenic microbes and earthworms than its linear form **6**, which is due to the fact that cyclization of peptides reduces the degree of freedom for each constituent within the ring and thus substantially leads to reduced flexibility, increased potency and selectivity of cyclic peptide. In contrast to synthetic heptacyclopeptide **7**, neither natural stylissamide G [19] nor other heptacyclopeptides of the stylissamide class isolated from the marine sponge *Stylissa caribica* including stylissamides A-F, H and X [19,27,59,60] are reported to possess pharmacological activity against pathogenic microbes and earthworms. However, literature supports inhibitory activity towards protein translation and cell migration possessed by stylissamide A and X respectively [26,27].

In comparison to other natural proline-rich cyclooligopeptides isolated from marine sponges which contain either two ‘Pro’ units adjacent to each other (e.g., hymenistatin 1, euryjanicin B [61,62]) or separated from each other by amino acid units such as tryptophan, histidine, serine or asparagine in the cyclic chain (e.g., stylissamide H, wainunamide, dominicin, axinellin A [19,63,64,65]), stylissamide G has a characteristic pattern of two ‘Pro’ units separated by one ‘Phe’ unit in a repeated manner in its structure. It is found to be associated with antibacterial, antifungal and anthelmintic effects, as supported by previous literature reports [5] involving cyclic peptide, viz. hymenamide E, which has antimicrobial and anthelmintic effects and the same type of ‘Pro’ and ‘Phe’ structural pattern as observed in heptacyclopeptide stylissamide G. 

The possible mechanism of action for the antimicrobial effect of heptacyclopeptide stylissamide G might involve the active transport inside the bacterial cell where it binds and inactivates specific targets such as the bacterial ribosome and thereby inhibits protein synthesis like other proline-rich antimicrobial peptides (PRAPs) [66]. This implies that PRAPs can be used as molecular hooks to identify the intracellular or membrane proteins that are involved in their mechanism of action and that may be subsequently used as targets for the design of novel antibiotics with mechanisms different from those now in use. Antifungal effects may be attributed to: the inhibition of chitin synthesis, a cell wall component essential to maintaining the structural integrity of the fungus; inhibition of 1–3 β glucan synthase, a multiunit membrane-integrated enzyme critical for cell wall integrity; transversion of the energized membrane and interaction with an intracellular target; and induction of reactive oxygen species (ROS) intracellularly that are toxic to the fungi, as reported for established anti-fungal peptides [67].

Heptacyclopeptide stylissamide G can be delivered intravenously or subcutaneously to avoid possible degradation and limited absorption in the gastrointestinal tract (GI). Although oral delivery is challenging, absorption enhancers, enzyme inhibitors, carrier systems and stability enhancers can be used to facilitate oral peptide delivery. Structural modification such as cyclization provides resistance to proteolytic degradation and has higher than expected absorption after oral administration in comparison to the linear counterparts. Additionally, delivering peptide transdermally allows the avoidance of both GI degradation and hepatic first-pass metabolism. Moreover, the intranasal route is another successful route for peptide drug delivery [68].

## 4. Materials and Methods

The melting point was determined by the open capillary method and is uncorrected. IR spectra were recorded using an FTIR-8400S Fourier transform spectrophotometer (Shimadzu, Kyoto, Japan). ^1^H NMR and ^13^C NMR spectra were recorded on a Bruker AC 300 spectrometer at 300 MHz (Brucker, Elk Grove Village, IL, USA). Mass spectra was recorded on a JMS-DX 303 spectrometer (Jeol, Tokyo, Japan). Elemental analysis was performed on a Vario EL III elemental analyzer (Elementar Vario EL III, Hanau, Germany) and optical rotation of the synthesized peptides was measured on an Optics Technology automatic polarimeter (OpticsTech, Delhi, India). Purity of the synthesized peptides was checked by thin layer chromatography (TLC) on precoated silica gel G plates (Kieselgel 0.25 mm, 60G F_254_, Merck, Germany).

### 4.1. General Procedure for the Synthesis of Linear Tetra/Tripeptide Segments *(**4**, **5**)*

To the solution of the amino acid methyl ester hydrochloride/dipeptide methyl ester (0.01 mol) in tetrahydrofuran (THF, 25 mL), NMM/TEA (2.23 mL/2.8 mL, 0.021 mol) was added at 0 °C, and the reaction mixture was stirred for 15 min. The Boc-protected dipeptide (0.01 mol) in THF (25 mL), (DIPC/EDC·HCl, 1.26 g/1.92 g, 0.01 mol) and HOBt (1.34 g, 0.01 mol) was added with stirring to the above reaction mixture. Stirring of the resulting mixture was continued for 24 h at room temperature (RT). The reaction mixture was filtered and the residue was washed with THF (25 mL) and added to the filtrate. The filtrate was washed with 5% NaHCO_3_ and saturated NaCl solutions. The organic layer was dried over anhydrous Na_2_SO_4_, filtered and evaporated in a vacuum. The crude product was recrystallized from a mixture of chloroform and petroleum ether (boiling point (b.p.) 40–60 °C) followed by cooling at 0 °C to obtain the title compounds.

*tert-Butyloxycarbonyl-l-phenylalanyl-l-prolyl-l-phenylalanyl-l-proline methyl ester* (**4**). Semisolid mass; Yield 87%; [α]_D_ = −73.7° (*c* = 0.25, MeOH); R*_f_* = 0.67 (CHCl_3_·MeOH-9:1); IR (CHCl_3_): *v* = 3127–3122 (N–H_str_, amide), 3069–3063 (Ar–H_str_, aromatic rings), 2998–2991 (C–H_str_, cyclic CH_2_), 2923, 2919 (C–H_str_, asym, CH_2_), 2843, 2837 (C–H_str_, sym, CH_2_), 1742 (C=O_str_, ester), 1667–1662, 1642–1638 (C=O_str_, 3° and 2° amide), 1566–1562, 1437–1433 (skeletal bands), 1539, 1532 (N–H_def_, amide), 1387, 1368 (C–H_def_, *tert*-butyl), 1271 (C–O_str_, ester), 716–712, 689–683 (C–H_def_, oop, aromatic rings) cm^−1^; ^1^H NMR (CDCl_3_): δ = 7.52, 7.47 (dd, *J* = 6.75, 4.5 Hz, 2H, *m*-H’s, Phe-1), 7.21, 7.16 (dd, *J* = 6.8, 4.45 Hz, 2H, *m*-H’s, Phe-2), 7.05 (t, *J* = 6.25 Hz, 1H, *p*-H, Phe-2), 6.92 (t, *J* = 6.3 Hz, 1H, *p*-H, Phe-1), 6.88–6.82 (m, 4 H, *o*-H’s, Phe-1 and Phe-2), 6.45 (br. s, 1H, N*H*, Phe-1), 6.38 (br. s, 1H, N*H*, Phe-2), 5.09 (q, *J* = 5.5 Hz, 1H, α-H, Phe-2), 4.56 (q, *J* = 5.45 Hz, 1H, α-H, Phe-1), 4.45 (t, *J* = 6.9 Hz, 1H, α-H, Pro-1), 3.92 (t, *J* = 6.85 Hz, 1H, α-H, Pro-2), 3.72 (t, *J* = 7.15 Hz, 2H, δ-H, Pro-1), 3.63 (s, 3 H, OC*H_3_*), 3.38 (t, *J* = 7.2 Hz, 2H, δ-H, Pro-2), 3.14 (d, *J* = 5.6 Hz, 2H, β-H’s, Phe-1), 2.92 (d, *J* = 5.55 Hz, 2H, β-H’s, Phe-2), 2.68–2.64 (m, 2H, β-H’s, Pro-1), 2.07–1.98 (m, 4 H, β-H’s, γ-H’s, Pro-2), 1.94–1.89 (m, 2H, γ-H’s, Pro-1), 1.52 (s, 9 H, *tert*-butyl); C_34_H_44_N_4_O_7_ (620): calcd. C 65.79, H 7.14, N 9.03; found C 65.76, H 7.12, N 9.05. 

*tert-Butyloxycarbonyl-l-leucyl-l-isoleucyl-l-proline methyl ester* (**5**). Semisolid mass; Yield 74%; [α]_D_ = +11.9° (*c* = 0.25, MeOH); R*_f_* = 0.49 (CHCl_3_·MeOH-9:1); IR (CHCl_3_): *v* = 3129, 3124 (N–H_str_, amide), 2999–2995 (C–H_str_, cyclic CH_2_), 2967, 2963–2958 (C–H_str_, asym, CH_3_), 2925 (C–H_str_, asym, CH_2_), 2856 (C–H_str_, sym, CH_2_), 1744 (C=O_str_, ester), 1666, 1643, 1639 (C=O_str_, 3° and 2° amide), 1536, 1532 (N–H_def_, 2° amide), 1388, 1367 (C–H_def_, *tert*-butyl), 1269 (C–O_str_, ester) cm^−1^; ^1^H NMR (CDCl_3_): δ = 7.05 (br. s, 1H, N*H*, Ile), 6.02 (br. s, 1H, N*H*, Leu), 4.36 (t, *J* = 8.55 Hz, 1H, α-H, Ile), 4.20 (q, *J* = 6.7 Hz, 1H, α-H, Leu), 3.92 (t, *J* = 6.85 Hz, 1H, α-H, Pro), 3.59 (s, 3 H, OC*H*_3_), 3.39 (t, *J* = 7.15 Hz, 2H, δ-H, Pro), 2.07–1.97 (m, 5 H, β-H’s, γ-H’s, Pro and β-H, Ile), 1.89 (t, *J* = 7.95 Hz, 2H, β-H’s, Leu), 1.67–1.62 (m, 2H, γ-H’s, Ile), 1.55 (s, 9 H, *tert*-butyl), 1.52–1.47 (m, 1H, γ-H, Leu), 1.04 (d, *J* = 5.85 Hz, 3 H, γ’-H’s, Ile), 0.99 (d, 6 H, *J* = 6.25 Hz, δ-H’s, Leu), 0.95 (t, 3 H, *J* = 7.8 Hz, δ-H’s, Ile); C_23_H_41_N_3_O_6_ (455): calcd. C 60.64, H 9.07, N 9.22; found C 60.63, H 9.10, N 9.25. 

### 4.2. Deprotection of the Tetrapeptide Unit *(**4**)* at the Amino Terminal

Boc-protected tetrapeptide (**4**, 6.21 g, 0.01 mol) was dissolved in CHCl_3_ (15 mL) and treated with CF_3_COOH (2.28 g, 0.02 mol). The resulting solution was stirred at room temperature for 1 h, and washed with a saturated NaHCO_3_ solution (25 mL). The organic layer was dried over anhydrous Na_2_SO_4_ and concentrated under reduced pressure. The crude product was purified by crystallization from CHCl_3_ and petroleum ether (b.p. 40–60 °C) to obtain the pure deprotected compound **4a**.

*l-Phenylalanyl-l-prolyl-l-phenylalanyl-l-proline methyl ester* (**4a**). Semisolid mass; Yield 80%; [α]_D_ = −38.4° (*c* = 0.25, MeOH); R*_f_* = 0.54 (CHCl_3_·MeOH-9:1); IR (CHCl_3_): *v* = 3128, 3125–3121 (N–H_str_, amide), 3066–3062 (Ar–H_str_, aromatic rings), 2999–2989 (C–H_str_, cyclic CH_2_), 2925, 2918 (C–H_str_, asym, CH_2_), 2842, 2835 (C–H_str_, sym, CH_2_), 1740 (C=O_str_, ester), 1666–1662, 1644–1639 (C=O_str_, 3° and 2° amide), 1565, 1435–1432 (skeletal bands), 1535, 1531 (N–H_def_, amide), 1270 (C–O_str_, ester), 715–711, 687–682 (C–H_def_, oop, aromatic rings) cm^−1^; ^1^H NMR (CDCl_3_): δ = 7.29, 7.25 (dd, *J* = 6.8, 4.45 Hz, 2H, *m*-H’s, Phe-1), 7.20, 7.14 (dd, *J* = 6.8, 4.5 Hz, 2H, *m*-H’s, Phe-2), 7.02 (t, *J* = 6.3 Hz, 1H, *p*-H, Phe-2), 6.95 (t, *J* = 6.25 Hz, 1H, *p*-H, Phe-1), 6.86, 6.83 (dd, *J* = 8.8, 4.15 Hz, 2H, *o*-H’s, Phe-2), 6.72, 6.69 (dd, *J* = 8.75, 4.2 Hz, 2H, *o*-H’s, Phe-1), 6.39 (br. s, 1H, N*H*, Phe-2), 5.07 (q, *J* = 5.45 Hz, 1H, α-H, Phe-2), 4.33 (t, *J* = 6.85 Hz, 1H, α-H, Pro-1), 3.95 (q, *J* = 5.45 Hz, 1H, α-H, Phe-1), 3.89 (t, *J* = 6.9 Hz, 1H, α-H, Pro-2), 3.61 (s, 3 H, OC*H_3_*), 3.54 (t, *J* = 7.15 Hz, 2H, δ-H, Pro-1), 3.39 (t, *J* = 7.2 Hz, 2H, δ-H, Pro-2), 2.95 (d, *J* = 5.6 Hz, 2H, β-H’s, Phe-2), 2.74 (d, *J* = 5.55 Hz, 2H, β-H’s, Phe-1), 2.69–2.65 (m, 2H, β-H’s, Pro-1), 2.24 (br. s, 2H, N*H_2_*, Phe-1), 2.06–1.97 (m, 4 H, β-H’s, γ-H’s, Pro-2), 1.93–1.88 (m, 2H, γ-H’s, Pro-1); C_29_H_36_N_4_O_5_ (520): calcd. C 66.90, H 6.97, N 10.76; found C 66.88, H 6.98, N 10.75.

### 4.3. Deprotection of the Tripeptide Unit *(**5**)* at the Carboxyl Terminal

To a solution of the tripeptide (**5**, 4.55 g, 0.01 mol) in THF·H_2_O (1:1, 36 mL) and LiOH (0.36 g, 0.015 mol) was added at 0 °C. The mixture was stirred at room temperature for 1 h and then acidified to pH = 3.5 with 1 N H_2_SO_4_. The aqueous layer was extracted with Et_2_O (3 × 25 mL). The combined organic extracts were dried over anhydrous Na_2_SO_4_ and concentrated under reduced pressure. The crude product was finally crystallized from methanol and ether to obtain the pure deprotected compound **5a**.

*tert-Butyloxycarbonyl-l-leucyl-l-isoleucyl-l-proline* (**5a**). Semisolid mass; Yield 71%; [α]_D_ = +41.5° (*c* = 0.25, MeOH); R*_f_* = 0.69 (CHCl_3_·MeOH-9:1); IR (CHCl_3_): *v* = 3296–2518 (O–H_str_, CO*OH*), 3129, 3125 (N–H_str_, amide), 2999–2994 (C–H_str_, cyclic CH_2_), 2965, 2962–2957 (C–H_str_, asym, CH_3_), 2922 (C–H_str_, asym, CH_2_), 2857 (C–H_str_, sym, CH_2_), 1710 (C=O_str_, *CO*OH), 1665, 1642, 1639 (C=O_str_, 3° and 2° amide), 1533, 1529 (N–H_def_, 2° amide), 1389, 1365 (C–H_def_, *tert*-butyl) cm^−1^; ^1^H NMR (CDCl_3_): δ = 10.49 (br. s, 1H, O*H*, COO*H*), 7.04 (br. s, 1H, N*H*, Ile), 6.06 (br. s, 1H, N*H*, Leu), 5.35 (t, *J* = 8.6 Hz, 1H, α-H, Ile), 4.18 (q, *J* = 6.65 Hz, 1H, α-H, Leu), 4.10 (t, *J* = 6.9 Hz, 1H, α-H, Pro), 3.38 (t, *J* = 7.2 Hz, 2H, δ-H, Pro), 2.05–1.94 (m, 5 H, β-H’s, γ-H’s, Pro and β-H, Ile), 1.87 (t, *J* = 8.0 Hz, 2H, β-H’s, Leu), 1.66–1.62 (m, 2H, γ-H’s, Ile), 1.54–1.49 (m, 1H, γ-H, Leu), 1.52 (s, 9 H, *tert*-butyl), 1.06 (d, *J* = 5.9 Hz, 3 H, γ’-H’s, Ile), 1.01 (d, 6 H, *J* = 6.3 Hz, δ-H’s, Leu), 0.96 (t, 3 H, *J* = 7.75 Hz, δ-H’s, Ile); C_22_H_39_N_3_O_6_ (441): calcd. C 59.84, H 8.90, N 9.52; found C 59.85, H 8.89, N 9.55. 

### 4.4. Procedure for the Synthesis of Linear Heptapeptide Unit and Its Cyclized form *(**6**, **7**)*

Tetrapeptide methyl ester, l-Phe-l-Pro-l-Phe-l-Pro-OMe (**4a**, 5.21 g, 0.01 mol) was dissolved in 30 mL of dichloromethane (DCM), and 2.23 mL/2.8 mL (0.021 mol) of TEA/NMM was added at 0 °C with the resulting mixture stirred for 15 min. Boc-protected tripeptide, Boc-l-Leu-l-Ile-l-Pro-OH (**5a**, 4.42 g, 0.01 mol) was dissolved in 30 mL of DCM and DIPC/EDC.HCl (1.26 g/1.92 g, 0.01 mol) and HOBt (1.34 g, 0.01 mol) was added to above mixture with stirring. Stirring continued for 24 h, after which the reaction mixture was filtered and the filtrate was washed with 25 mL each of 5% NaHCO_3_ and saturated NaCl solutions. The organic layer was dried over anhydrous Na_2_SO_4_, filtered and evaporated in vacuum. The crude product was recrystallized from a mixture of chloroform and petroleum ether (b.p. 40–60 °C) followed by cooling at 0 °C to obtain the Boc-l-Leu-l-Ile-l-Pro-l-Phe-l-Pro-l-Phe-l-Pro-OMe (**6**) as a yellowish semisolid mass. The linear heptapeptide unit (**6**, 4.72 g, 0.005 mol) was then deprotected at the carboxyl terminal using lithium hydroxide (LiOH, 0.18 g, 0.0075 mol) to obtain the Boc-l-Leu-l-Ile-l-Pro-l-Phe-l-Pro-l-Phe-l-Pro-OH. To a solution of the deprotected heptapeptide (4.65 g, 0.005 mol) in CHCl_3_ (50 mL), pentafluorophenol (*pfp*, 1.23 g, 0.0067 mol) and dicyclohexylcarbodiimide (DCC, 1.06 g, 0.005 mol) were added, followed by stirring at RT for 12 h. Filtrate of the above reaction mixture was washed with 10% NaHCO_3_ (3 × 20 mL) and 5% HCl (2 × 20 mL) solutions to obtain the corresponding pentafluorophenyl ester Boc-l-Leu-l-Ile-l-Pro-l-Phe-l-Pro-l-Phe-l-Pro-O*pfp*. The Boc group of the resulting unit (4.38 g, 0.004 mol) was removed using CF_3_COOH (0.91 g, 0.008 mol) to obtain the deprotected product l-Leu-l-Ile-l-Pro-l-Phe-l-Pro-l-Phe-l-Pro-O*pfp,* which was dissolved in CHCl_3_ (25 mL), and TEA/NMM/pyridine (2.8 mL/2.21 mL/1.61 mL, 0.021 mol) was added. Then, the entire contents were kept at 0 °C for 7 days. The reaction mixture was washed with 10% NaHCO_3_ (3 × 25 mL) and 5% HCl (2 × 25 mL) solutions. The organic layer was dried over anhydrous Na_2_SO_4_ and crude cyclized compound was recrystallized from CH_2_Cl_2_/*n*-hexane to obtain the pure product *cyclo*(l-leucyl-l-isoleucyl-l-prolyl-l-phenylalanyl-l-prolyl-l-phenylalanyl-l-prolyl) (**7**).

*tert-Butyloxycarbonyl-l-leucyl-l-isoleucyl-l-prolyl-l-phenylalanyl-l-prolyl-l-phenylalanyl-l-proline methyl ester* (**6**). Semisolid mass; Yield 89%; [α]_D_ = −112.4° (*c* = 0.1, MeOH); R*_f_* = 0.68 (CHCl_3_·MeOH-8:2); IR (CHCl_3_): *v* = 3129, 3126–3122 (N–H_str_, amide), 3067–3062 (Ar–H_str_, aromatic rings), 2999–2992 (C–H_str_, cyclic CH_2_), 2965, 2961–2957 (C–H_str_, asym, CH_3_), 2924–2919 (C–H_str_, asym, CH_2_), 2842, 2839 (C–H_str_, sym, CH_2_), 1745 (C=O_str_, ester), 1669–1664, 1644–1639 (C=O_str_, 3° and 2° amide), 1568, 1562, 1437–1433 (skeletal bands), 1539–1531 (N–H_def_, amide), 1387, 1369 (C–H_def_, *tert*-butyl), 1273 (C–O_str_, ester), 715–712, 687–682 (C–H_def_, oop, aromatic rings) cm^−1^; ^1^H NMR (CDCl_3_): δ = 7.28, 7.24 (dd, *J* = 6.8, 4.5 Hz, 2H, *m*-H’s, Phe-1), 7.20, 7.16 (dd, *J* = 6.75, 4.45 Hz, 2H, *m*-H’s, Phe-2), 7.09 (br. s, 1H, N*H*, Ile), 7.03 (t, *J* = 6.3 Hz, 1H, *p*-H, Phe-2), 6.97 (t, *J* = 6.25 Hz, 1H, *p*-H, Phe-1), 6.85–6.81 (m, 4 H, *o*-H’s, Phe-1 and Phe-2), 6.42 (br. s, 1H, N*H*, Phe-1), 6.38 (br. s, 1H, N*H*, Phe-2), 6.05 (br. s, 1H, N*H*, Leu), 5.07 (q, *J* = 5.45 Hz, 1H, α-H, Phe-2), 4.89 (q, *J* = 5.5 Hz, 1H, α-H, Phe-1), 4.58 (t, *J* = 8.6 Hz, 1H, α-H, Ile), 4.19 (q, *J* = 6.7 Hz, 1H, α-H, Leu), 4.13 (t, *J* = 6.85 Hz, 1H, α-H, Pro-1), 3.93 (t, *J* = 6.9 Hz, 1H, α-H, Pro-2), 3.89 (t, *J* = 6.85 Hz, 1H, α-H, Pro-3), 3.62 (s, 3 H, OC*H_3_*), 3.39 (t, *J* = 7.15 Hz, 2H, δ-H, Pro-3), 3.34–3.29 (m, 4 H, δ-H, Pro-2 and Pro-1), 2.94 (d, *J* = 5.6 Hz, 2H, β-H’s, Phe-2), 2.76 (d, *J* = 5.55 Hz, 2H, β-H’s, Phe-1), 2.69–2.63 (m, 4 H, β-H’s, Pro-1 and Pro-2), 2.07–1.96 (m, 5 H, β-H’s, γ-H’s, Pro-3 and β-H, Ile), 1.95–1.90 (m, 4 H, γ-H’s, Pro-2 and Pro-1), 1.87 (t, *J* = 8.0 Hz, 2H, β-H’s, Leu), 1.67–1.62 (m, 2H, γ-H’s, Ile), 1.55 (s, 9 H, *tert*-butyl), 1.53–1.49 (m, 1H, γ-H, Leu), 1.03 (d, *J* = 5.9 Hz, 3 H, γ’-H’s, Ile), 0.98 (d, 6 H, *J* = 6.3 Hz, δ-H’s, Leu), 0.96 (t, 3 H, *J* = 7.75 Hz, δ-H’s, Ile); ^13^C NMR (CDCl_3_): δ = 173.5 (C=O, Leu), 173.1 (C=O, Pro-2), 172.5 (C=O, Pro-1), 170.4 (C=O, Phe-1), 168.8 (C=O, Pro-3), 167.9 (C=O, Ile), 167.1 (C=O, Phe-2), 153.4 (C=O, Boc), 136.1 (γ-C, Phe-1), 134.4 (γ-C, Phe-2), 131.9 (2 C, *o*-C’s, Phe-2), 129.8 (2 C, *o*-C’s, Phe-1), 129.3 (2 C, *m*-C’s, Phe-2), 128.7 (2 C, *m*-C’s, Phe-1), 128.2 (*p*-C, Phe-1), 127.4 (*p*-C, Phe-2), 79.6 (α-C, Boc), 58.9 (α-C, Pro-3), 55.3 (α-C, Pro-2), 54.1 (α-C, Pro-1), 53.3 (O*C*H_3_), 52.6, 49.4 (2 C, α-C’s, Leu and Ile), 48.1, 47.4 (2 C, α-C’s, Phe-2 and Phe-1), 46.8, 46.1, 45.5 (3 C, δ-C’s, Pro-1, Pro-2 and Pro-3); 39.9 (β-C, Leu), 37.4 (β-C, Phe-2), 36.5, 35.1 (2 C, β-C, Phe-1 and Ile), 28.9 (β-C, Pro-3), 28.2 (3 C, β-C’s, Boc), 27.7, 26.9 (2 C, β-C, Pro-2 and Pro-1), 25.4, 24.9 (2 C, γ-C’s, Ile and Pro-3), 24.4, 23.8 (2 C, γ-C’s, Pro-2 and Pro-1), 23.6 (2 C, δ-C’s, Leu), 22.0 (γ-C, Leu), 17.4 (γ’-C, Ile), 10.1 (δ-C, Ile); C_51_H_73_N_7_O_10_ (944): calcd. C 64.88, H 7.79, N 10.38; found C 64.89, H 7.82, N 10.36. 

*Cyclo (l-leucyl-l-isoleucyl-l-prolyl-l-phenylalanyl-l-prolyl-l-phenylalanyl-l-prolyl)* (**7**). White solid; m.p. 121–123 °C (d); Yield 86% (C_5_H_5_N), 77% (NMM), 72% (TEA); [α]_D_ = −93.1° (*c* = 0.1, MeOH); R*_f_* = 0.81 (CHCl_3_·MeOH-8:2); IR (KBr): *v* = 3128, 3125–3119 (N–H_str_, amide), 3068–3063 (Ar–H_str_, aromatic rings), 2999, 2996–2989 (C–H_str_, cyclic CH_2_), 2968, 2962–2956 (C–H_str_, asym, CH_3_), 2925–2919 (C–H_str_, asym, CH_2_), 2844, 2838 (C–H_str_, sym, CH_2_), 1668–1663, 1646–1639 (C=O_str_, 3° and 2° amide), 1567, 1560, 1439–1432 (skeletal bands), 1535–1529 (N–H_def_, amide), 717–711, 689–683 (C–H_def_, oop, aromatic rings) cm^−1^; ^1^H NMR (CDCl_3_): δ = 9.75 (br. s, 1H, N*H*, Phe-2), 9.74 (br. s, 1H, N*H*, Phe-1), 9.32 (br. s, 1H, N*H*, Leu), 9.05 (br. s, 1H, N*H*, Ile), 7.23, 7.19 (dd, *J* = 6.75, 4.5 Hz, 2H, *m*-H’s, Phe-1), 7.17, 7.14 (dd, *J* = 6.8, 4.45 Hz, 2H, *m*-H’s, Phe-2), 7.02 (t, *J* = 6.25 Hz, 1H, *p*-H, Phe-2), 6.98 (t, *J* = 6.3 Hz, 1H, *p*-H, Phe-1), 6.84–6.79 (m, 4 H, *o*-H’s, Phe-1 and Phe-2), 5.25 (t, *J* = 8.55 Hz, 1H, α-H, Ile), 5.09 (q, *J* = 6.65 Hz, 1H, α-H, Leu), 4.41 (q, *J* = 5.5 Hz, 1H, α-H, Phe-2), 3.36 (q, *J* = 5.45 Hz, 1H, α-H, Phe-1), 3.94 (t, *J* = 6.9 Hz, 1H, α-H, Pro-1), 3.91 (t, *J* = 6.85 Hz, 1H, α-H, Pro-2), 3.88 (t, *J* = 6.9 Hz, 1H, α-H, Pro-3), 3.28 (t, *J* = 7.15 Hz, 2H, δ-H’s, Pro-3), 3.24–3.19 (m, 4 H, δ-H’s, Pro-2 and Pro-1), 2.69–2.62 (m, 6 H, β-H’s, Pro-1, Pro-3 and Pro-2), 2.54 (d, *J* = 5.6 Hz, 2H, β-H’s, Phe-2), 2.48 (d, *J* = 5.55 Hz, 2H, β-H’s, Phe-1), 1.88 (t, *J* = 7.95 Hz, 2H, β-H’s, Leu), 1.84–1.79 (m, 4 H, γ-H’s, Pro-2 and Pro-1), 1.78–1.73 (m, 2H, γ-H’s, Pro-3), 1.64–1.59 (m, 2H, γ-H’s, Ile), 1.49–1.45 (m, 1H, β-H’s, Ile), 1.01 (d, 6 H, *J* = 6.25 Hz, δ-H’s, Leu), 0.99 (d, *J* = 5.9 Hz, 3 H, γ’-H’s, Ile), 0.95 (t, 3 H, *J* = 7.8 Hz, δ-H’s, Ile), 0.86–0.79 (m, 1H, γ-H, Leu); ^13^C NMR (CDCl_3_): δ = 173.9 (C=O, Pro-2), 171.4 (C=O, Ile), 171.1 (C=O, Pro-3), 170.5 (C=O, Pro-1), 169.8 (C=O, Leu), 169.2 (C=O, Phe-1), 168.5 (C=O, Phe-2), 139.9 (γ-C, Phe-1), 139.2 (γ-C, Phe-2), 130.6 (2 C, *o*-C’s, Phe-2), 129.9 (2 C, *o*-C’s, Phe-1), 129.5 (2 C, *m*-C’s, Phe-2), 128.9 (2 C, *m*-C’s, Phe-1), 127.8 (*p*-C, Phe-1), 127.2 (*p*-C, Phe-2), 59.5 (α-C, Pro-3), 58.4 (α-C, Pro-2), 56.6 (α-C, Pro-1), 56.2, 55.6 (2 C, α-C’s, Leu and Ile), 51.7, 51.2 (2 C, α-C’s, Phe-2 and Phe-1), 50.2, 48.8, 46.9 (3 C, δ-C’s, Pro-1, Pro-2 and Pro-3); 42.6 (β-C, Leu), 38.8 (β-C, Phe-2), 37.4, 36.4 (2 C, β-C, Phe-1 and Ile), 34.7 (β-C, Pro-3), 32.8, 31.2 (2 C, β-C, Pro-2 and Pro-1), 27.3, 24.0 (2 C, γ-C’s, Leu and Ile), 22.5 (2 C, δ-C’s, Leu), 21.7, 20.1, 19.6 (3 C, γ-C’s, Pro-2, Pro-3 and Pro-1), 16.8 (γ’-C, Ile), 10.4 (δ-C, Ile); MS (FAB, 70 eV): *m/z* (%) = 813 (100) [M + 1]^+^, 785 (14) [813-CO]^+^, 715 (65) [Leu-Ile-Pro-Phe-Pro-Phe]^+^, 699 (39) [Ile-Pro-Phe-Pro-Phe-Pro]^+^, 687 (11) [715-CO]^+^, 671 (10) [699-CO]^+^, 665 (77) [Pro-Leu-Ile-Pro-Phe-Pro]^+^, 637 (13) [665-CO]^+^, 602 (70) [Ile-Pro-Phe-Pro-Phe]^+^, 586 (56) [Pro-Phe-Pro-Phe-Pro]^+^, 574 (16) [602-CO]^+^, 568 (47) [Leu-Ile-Pro-Phe-Pro]^+^, 558 (11) [586-CO]^+^, 540 (16) [568-CO]^+^, 489 (44) [Phe-Pro-Phe-Pro]^+^, 471 (59) [Leu-Ile-Pro-Phe]^+^, 461 (15) [489-CO]^+^, 455 (78) [Ile-Pro-Phe-Pro]^+^, 443 (11) [471-CO]^+^, 427 (14) [455-CO]^+^, 421 (39) [Pro-Leu-Ile-Pro]^+^, 393 (10) [421-CO]^+^, 392 (55) [Phe-Pro-Phe]^+^, 364 (14) [392-CO]^+^, 358 (37) [Ile-Pro-Phe]^+^, 342 (46) [Pro-Phe-Pro]^+^, 324 (28) [Pro-Leu-Ile]^+^, 330 (11) [358-CO]^+^, 324 (29) [Pro-Leu-Ile]^+^, 314 (16) [342-CO]^+^, 296 (10) [324-CO]^+^, 245 (26) [Pro-Phe]^+^, 227 (22) [Leu-Ile]^+^, 217 (11) [245-CO]^+^, 211 (29) [Pro-Leu]^+^, 199 (11) [227-CO]^+^, 183 (10) [211-CO]^+^, 148 (21) [Phe]^+^, 120 (28) [Phe immonium ion, C_8_H_10_N]^+^, 114 (14) [Ile]^+^, 98 (11) [Pro]^+^, 91 (17) [C_7_H_7_]^+^, 86 (19) [Leu/Ile immonium ion, C_5_H_12_N]^+^, 77 (15) [C_6_H_5_]^+^, 70 (39) [Pro immonium ion, C_4_H_8_N]^+^, 57 (10) [C_4_H_9_]^+^, 43 (15) [C_3_H_7_]^+^, 29 (12) [C_2_H_5_]^+^, 15 (17) [CH_3_]^+^; C_45_H_61_N_7_O_7_ (812): calcd. C 66.56, H 7.57, N 12.07; found C 66.58, H 7.56, N 12.05. 

### 4.5. Biological Evaluation

#### 4.5.1. Anthelmintic Screening

Anthelmintic activity studies for the newly synthesized linear and cyclic heptapeptides (**6**, **7**) were carried out for the three different species of the earthworms *M. konkanensis*, *P. corethruses* and *E. eugeniea* at a 2 mg/mL concentration. Suspensions of the samples were prepared by triturating the synthesized compounds (100 mg) with Tween 80 (0.5%) and distilled water and the resulting mixtures were stirred using a mechanical stirrer for 30 min. The suspensions were diluted to contain 0.2% *w/v* of the test samples. Suspensions of the reference drug, mebendazole were prepared with the same concentration in a similar way. Three sets of the five earthworms of almost similar sizes (2 inches in length) were placed in the Petri plates of 4-inch diameter containing 50 mL of the suspension of test sample and the reference drug at RT. Another set of five earthworms was kept as control in 50 mL suspension of the distilled water and Tween 80 (0.5%). The paralyzing and death times were noted and their mean was calculated for the triplicate sets. The death time was ascertained by placing the earthworms in the warm water (50 °C) which stimulated movement if the worm was alive. The results of the anthelmintic studies are tabulated in Table 1.

#### 4.5.2. Antibacterial Screening

The newly synthesized linear and cyclic heptapeptides (**6**, **7**) were evaluated for their antibacterial potential against the two Gram-positive bacteria, *Bacillus subtilis*, *Staphylococcus aureus* and two Gram-negative bacteria, *Pseudomonas aeruginosa* and *Klebsiella pneumoniae*, at concentrations of 50–6.25 μg/mL. MIC values of test compounds were determined by the tube dilution technique. Both linear and the cyclic heptapeptides were dissolved separately to prepare a stock solution of 1 mg/mL using DMF. The stock solution was aseptically transferred and suitably diluted with the sterile broth medium to contain seven different concentrations of each test compound ranging from 200 to 3.1 μg/mL in different test tubes. All the tubes were inoculated with one loopful of one of the test bacteria. The process was repeated with the different test bacteria and the different samples. The tubes inoculated with bacterial cultures were incubated at 37 °C for 18 h and the presence/absence of growth of the bacteria was observed. From these results, the MIC of each test compound was determined against each test bacterium. A possible spore suspension was prepared in sterile distilled water from a 5-day-old culture of the test bacteria growing on nutrient broth media. About 20 mL of the growth medium was transferred into the sterilized Petri plates and inoculated with 1.5 mL of the spore suspension (spore concentration ~ 6 × 10^4^ spores/mL). Filter paper disks 6 mm in diameter and 1 mm in thickness were sterilized by autoclaving at 121 °C (15 psi) for 15 min. Each Petri plate was divided into five equal portions along the diameter to place one disc. Three discs of the test sample were placed on three portions together with one disc with the reference drug gatifloxacin, and a disk impregnated with the solvent (DMF) as the negative control. The Petri plates inoculated with bacterial cultures were incubated at 37 °C for 18 h. Diameters of the inhibition zones (in mm) were measured and the average diameters for the test sample were calculated in triplicate. The diameters obtained for the test sample were compared with that produced by the standard drug. The results of the antibacterial studies are presented in Table 2.

#### 4.5.3. Antifungal Screening

The serial plate dilution method was used for the evaluation of antifungal activity against the diamorphic fungal strain *C. albicans* and three other fungal strains, including *A. niger* and two cutaneous fungal strains, *M. audouinii* and *T. mentagrophytes,* at the concentrations of 50–6.25 μg/mL for the newly synthesized linear and cyclic heptapeptides (**6**, **7**). MIC values of the test compounds were determined by employing the same technique as used for the antibacterial studies using DMSO instead of DMF and tubes inoculated with fungal cultures were incubated at 37 °C for 48 h. After incubation, the presence/absence of the fungal growth was observed and MIC of the test compounds was determined against each test fungus. A spore suspension in the normal saline (0.91% *w/v* of NaCl) was prepared from the culture of the test fungi on Sabouraud’s broth media. After transferring the growth medium, the Petri plates were inoculated with the spore suspension. After drying, wells were made using an agar punch and test samples; the reference drug griseofulvin and negative control (DMSO) were placed in the labeled wells in each Petri plate. The Petri plates inoculated with the fungal cultures were incubated at 37 °C for 48 h. Antifungal activity was determined by measuring the diameter of the inhibition zone for the triplicate sets. The activity of each compound was compared with the reference standard. The results of the antifungal studies are given in the Table 2.

Experimental details of the biological activity studies are described in our earlier reports [69,70,71,72,73]. Further, in order to describe the intermolecular forces of drug receptor interaction as well as transport and distribution of drugs in a quantitative manner, various steric and the lipophilicity parameters needed to be calculated for the synthesized linear and cyclic heptapeptide (**6**, **7**) (Table S1). As per IUPAC rules, the heptacyclopeptide **7** can be named as “17,25-Dibenzyl-6-(*sec*-butyl)-9-isobutylperhydrotripyrrolo[1,2-*a*:1,2-*g*:1.2-*m*][1,4,7,10,13,16,19]heptaaza cyclohenicosine-5,8,11,16,19, 24,27-heptaone”.

## 5. Conclusions

An efficient strategy was developed toward the first total synthesis of the natural cyclopolypeptide stylissamide G (**7**) and for the preparation of its unusual linear tetrapeptide, tripeptide and heptapeptide (**4**–**6**) building blocks via coupling reactions utilizing carbodiimide chemistry in the alkaline environment in the presence of racemization suppressing agent. The DIPC/NMM coupling method proved to be the yield-effective in comparison to the methods utilizing EDC·HCl/DIPC and TEA, providing 10%–12% additional yield. The pentafluorophenyl ester was shown to be better for the activation of the acid functionality of the linear heptapeptide unit. Pyridine was found to be a good base for the intramolecular cyclization of the linear peptide fragment in comparison to TEA or NMM. Like other proline-containing synthetic cyclic heptapeptides (e.g., hymenamide E, segetalin E, or gypsin D), the newly synthesized heptacyclopeptide displayed potent anthelmintic activity against earthworms *M. konkanensis*, *P. corethruses* and *E. eugeniea* and effectiveness against pathogenic dermatophtytes *M. audouinii*, *T. mentagrophytes* and *C. albicans*. Synthesized cyclic peptides bearing good bioactivity against earthworms may prove to be future anthelmintic candidates for treating parasitic worm infections, where resistance to established drugs is the prime target of focus. In addition, Gram-negative bacteria *P. aeruginosa* and *K. pneumoniae* were found to be more sensitive than Gram-positive bacteria *B. subtilis* and *S. aureus* to the newly synthesized peptide. The newly synthesized heptacyclopeptide might act through active transportation inside the bacterial cell and inhibition of the protein synthesis by binding and inactivating the bacterial ribosome. Antifungal action of the cycloheptapeptide might be attributed to the inhibition of glucan/cell wall chitin/sphingolipids synthesis. On passing toxicity tests, heptacyclopeptide **7** may prove as a good candidate for the clinical studies and could be a new antifungal and anthelmintic drug of the future.

## Figures and Tables

**Table 1:** Anthelmintic screening data for the linear and cyclic heptapeptides (**6**, **7**).

Compound	Earthworm Species					
	*M. konk.*		*P. core.*		*E. euge.*	
	Mean Paralyzing Time (min) ^‡^	Mean Death Time (min) ^‡^	Mean Paralyzing Time (min)	Mean Death Time (min)	Mean Paralyzing Time (min)	Mean Death Time (min)
**6**	13.50 ± 0.11	21.52 ± 0.40	17.21 ± 0.22	27.55 ± 0.26	12.48 ± 0.44	23.28 ± 0.17
**7**	09.13 ± 0.31	15.48 ± 0.52	12.55 ± 0.37	21.27 ± 0.19	09.25 ± 0.35	18.09 ± 0.22
Control ^#^	–	–	–	–	–	–
Mebendazole	13.63 ± 0.30	22.43 ± 0.21	17.56 ± 0.43	29.49 ± 0.17	13.50 ± 0.34	24.07 ± 0.49

*M. konk.*: *Megascoplex konkanensis*; *P. core.*: *Pontoscotex corethruses*; *E. euge.*: *Eudrilus eugeniea*; **^‡^** Data are given as mean ± S.D. (*n* = 3); ^#^ Tween 80 (0.5%) in distilled water.

**Table 2:** Antimicrobial screening data for linear and cyclic heptapeptides (**6**, **7**).

Compound	Diameter of Zone of Inhibition (mm)							
	Bacterial Strains				Fungal Strains			
	*B. sub.*	*S. aur.*	*P. aeru.*	*K. pneu.*	*C. alb.*	*M. audo.*	*A. niger*	*T. menta.*
**6**	–	–	14(6)	19(6)	17(6)	19(6)	–	18(6)
**7**	10(25)	11(12.5)	18(6)	22(6)	22(6)	23(6)	–	22(6)
Control *	–	–	–	–	–	–	–	–
Gatifloxacin	18(12.5) ^†^	27(6)	23(6)	25(6)	–	–	–	–
Griseofulvin	–	–	–	–	20(6)	18(6)	20(12.5)	19(6)

*B. sub.*: *Bacillus subtilus*; *S. aur.*: *Staphylococcus aureus*; *P. aeru.*: *Pseudomonas aeruginosa*; *K. pneu.*: *Klebsiella pneumonia*; *C. alb.*: *Candida albicans*; *M. audo.*: *Microsporum audouinii*; *A. niger: Aspergillus niger*; *T. menta.*: *Trichophyon mentagrophytes*; ^†^ Values in bracket are minimum inhibitory concentrations (MIC) values (μg/mL); * Dimethylformamide (DMF)/Dimethyl sulfoxide (DMSO).

## Supplementary Materials

The following are available online at www.mdpi.com/1660-3397/14/12/228/s1, Figure S1: Mass fragmentation pattern for heptacyclopeptide **7** at different amide bond levels, Table S1: Various steric and lipophilicity parameters for linear and cyclic peptides (**6**, **7**).
