# Supplementary material for: First Total Synthesis and Biological Screening of a Proline-Rich Cyclopeptide from a Caribbean Marine Sponge
Source: Mar Drugs. 2016 Dec 15;14(12):228. doi: 10.3390/md14120228 (PMC5192465; doi:10.3390/md14120228)
Supplement: Supplementary file 1 [file marinedrugs-14-00228-s001.doc]

Supplementary Materials: First Total Synthesis and Biological Screening of a Proline-Rich Cyclopeptide from a Caribbean Marine Sponge

Rajiv Dahiya, Sunil Singh, Ajay Sharma, Suresh V. Chennupati and Sandeep Maharaj


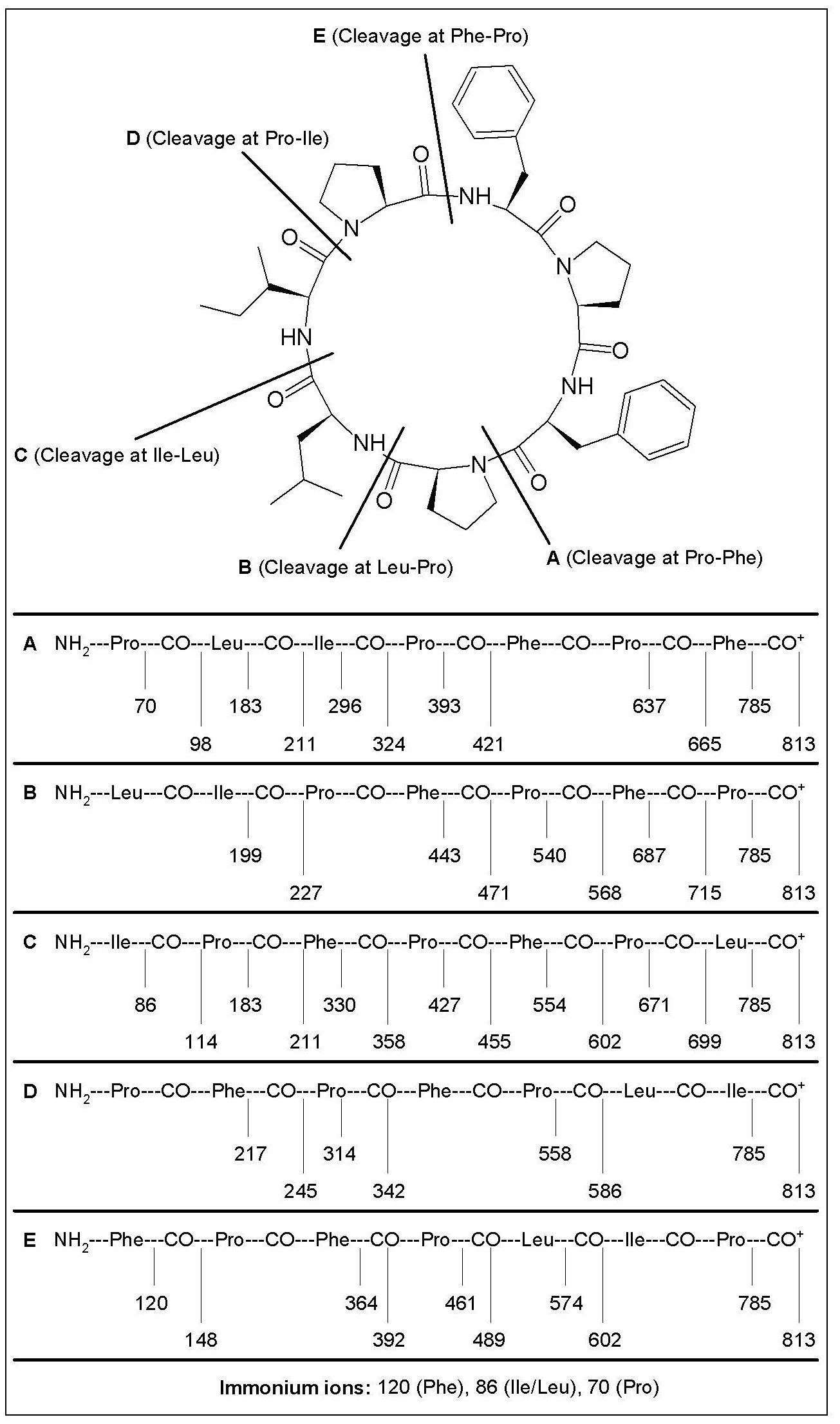


**Figure S1****.** Mass fragmentation pattern for heptacyclopeptide **7** at different amide bond levels.

**Table S1.** Various steric and lipophilicity parameters for linear and cyclic peptides (**6**, **7**).

| **Parameter** | *** Calculated Value for** | |
| --- | --- | --- |
|  | **Compound 6** | **Compound 7** |
| Molar Refractivity (MR^20^) | 253.28 ± 0.3 cm^3^ | 222.97 ± 0.4 cm^3^ |
| Molar Volume (MV^20^) | 783.3 ± 3.0 cm^3^ | 639.1 ± 5.0 cm^3^ |
| Parachor (P_r_) | 2103.7 ± 6.0 cm^3^ | 1791.7 ± 6.0 cm^3^ |
| Refractive Index (n^20^) | 1.560 ± 0.02 | 1.614 ± 0.03 |
| Surface Tension (γ^20^) | 52.0 ± 3.0 dyne/cm | 61.7 ± 5.0 dyne/cm |
| Density (d^20^) | 1.205 ± 0.06 g·cm^−3^ | 1.27 ± 0.1 g·cm^−3^ |
| Polarizability (α) | 100.41 ± 0.5 × 10^−24^ cm^3^ | 88.39 ± 0.5 × 10^−24^ cm^3^ |
| log P (*n*-Octanol/water) | 9.33 ± 0.91 | −0.78 ± 0.94 |

* Values were calculated using ACD/ChemSketch 2.0 software.
